# Relaxin Does Not Improve Angiotensin II-Induced Target-Organ Damage

**DOI:** 10.1371/journal.pone.0093743

**Published:** 2014-04-07

**Authors:** Nadine Haase, Julianna Rugor, Lukasz Przybyl, Fatimunnisa Qadri, Dominik N. Müller, Ralf Dechend

**Affiliations:** 1 Experimental and Clinical Research Center, a joint cooperation between the Max-Delbrueck Center for Molecular Medicine and the Charité Medical Faculty, Berlin, Germany; 2 Department of Cardiology and Nephrology, HELIOS-Klinikum Berlin, Berlin, Germany; Universtiy of Maryland Schoool of Medicine, United States of America

## Abstract

Relaxin is a corpus-luteum produced protein hormone with vasodilatatory, anti-fibrotic, and angiogenic properties that are opposite to angiotensin (Ang) II. We investigated whether or not relaxin ameliorates Ang II-induced target-organ damage. We used double transgenic rats harboring both human renin and angiotensinogen genes (dTGR) that develop severe hypertension, target-organ damage, and die untreated within 7–8 weeks. Recombinant relaxin at a low (26 μg/kg/d) and a high dose (240 μg/kg/d) was given to 4 week-old dTGR and age-matched Sprague-Dawley rats (SD). Systolic blood pressure increased progressively in untreated dTGRs from 162±3 mmHg at week 5 to 225±5 mmHg at week 7. Relaxin had no effect on blood pressure whereas SD rats were normotensive (106±1 mmHg). Untreated and relaxin-treated dTGR had similarly severe cardiac hypertrophy indices. Relaxin did not ameliorate albuminuria and did not prevent matrix-protein deposition in the heart and kidney in dTGR. Finally, relaxin treatment did not reduce mortality. These data suggest that pharmacological doses of relaxin do not reverse severe effects of Ang II.

## Introduction

Relaxin is a small peptide hormone important in reproduction and pregnancy that is encoded by the *RLX* gene [1]. During pregnancy, relaxin is produced by the corpus luteum; the hormone reaches a peak plasma concentration in the late first trimester and at delivery. Relaxin mediates the hemodynamic changes that occur during pregnancy, such as increased cardiac output, increased renal blood flow, and increased arterial compliance. Relaxin also relaxes pelvic ligaments and is believed to soften the pubic symphysis. Relaxin has anti-inflammatory, anti-apoptotic, vasodilatory, and anti-fibrotic properties [2]–[4]. Male *RLX* gene-deficient mice showed cardiac fibrosis, ventricular stiffening, and diastolic dysfunction, suggesting a protective role for relaxin in these processes [5]. Relaxin also increases arterial compliance, cardiac output, and renal blood flow, which are potentially relevant to the treatment of acute heart failure [2]–[4]. In two recent studies, a 48 h relaxin infusion in patients with acute heart failure showed beneficial effects on post-discharge mortality [6], [7]. A potential role for relaxin in protecting from preeclampsia and the implication that upregulation of the renin-angiotensin system could play a role in that condition caused us to test the hypothesis that relaxin could ameliorate Ang II-induced target-organ damage[8]–[11]. We used the well-established double transgenic human-angiotensinogen and human-renin rat model (dTGR) [12]–[15]. At age 7 weeks, dTGR show striking cardiac hypertrophy with fibrosis, severe diastolic dysfunction but preserved systolic function, proteinuria, and renal fibrosis [12]–[15]. Large areas of infarction are absent, markers of critical ischemia are negative, only rare patchy areas of myocardial necrosis can be observed in dTGR^6^. Ang II induces a sustained inflammatory response, which is to a large extending responsible for the severe phenotype. Blocking the Renin-Angiotensin System is very successful approach in ameliorating target-organ damage in this model [16], but several anti-inflammatory strategies, such as high dose aspirin, TNF-receptor blocker, steroids, MMF and statins reduced end-organ damage often independent of reducing blood pressure [14], [17]–[19]. Thus we investigated whether relaxin is reducing hypertensive target-organ damage in dTGR

## Methods

Experiments were conducted in 4 week-old male age-matched and body weight-matched transgenic rats harboring human renin and angiotensinogen genes (dTGR) (Harlan, Netherlands) and nontransgenic Sprague-Dawley rats (SD, Janvier, France). Local authorities approved the studies (permit number: G0015/13) and all procedures were done according to guidelines from the American Physiological Society. All surgery was performed under isoflurane anesthesia, and all efforts were made to minimize suffering. Human recombinant relaxin was kindly provided by Novartis, Switzerland and has been shown previously to be bioactive in rodents [20].

We compared untreated dTGR receiving vehicle (20 mM sodium acetate, pH 5.0, n = 14), low dosage of relaxin (26 μg/kg per day, n = 13), high dosage of relaxin (240 μg/kg per day, n = 13), and SD control rats receiving vehicle (20 mM sodium acetate, pH 5.0, n = 8) by subcutaneous osmotic minipump for 3 weeks. Treatments began when the rats were 4 weeks of age. Systolic blood pressure was measured at week 5,6 and 7 by tail cuff. Twenty-four-hour urine samples were collected in metabolic cages also at week 5, 6 and 7. Urinary rat albumin was measured with a commercially available ELISA (CellTrend, Germany). Serum concentration of relaxin was also measured with a commercially available ELISA (Immundiagnostik, Germany). Serum cystatin C was measured with a commercially available ELISA (BioVendor, Germany) and serum creatinine were determined by an automated clinical method.

Rats were killed at age of 7 weeks. The kidneys and hearts were washed with ice cold saline, blotted dry, and weighed. For gene expression analysis, the tissues were snap-frozen in liquid nitrogen and stored at −80°C. For immuno- and histochemistry, the tissues were formalin fixed and paraffin embedded. Paraffin-embedded sections were cut 4-6 μm thick and stained for collagens in heart sections with Sirius red and in kidney sections with Masson's trichrome as described earlier [21], [22]. Kindney sections were incubated with primary monoclonal antibodies against rat monocytes/macrophages (ED-1, 1∶500; Serotec), and with Cy3 labelled secondary antibody against anti mouse (1∶100; Jackson Immuno Research). Semiquantitative scoring of ED-1-positive cells was performed with use of a computerized cell count program and described before in detail [18].

For quantitative RT-PCR, total mRNA was isolated with the Qiagen RNeasy mini Kit (including the RNase-Free DNase set; Qiagen) according to the manufacturer's protocol from hearts and kidneys of 7 week-old SD and dTGR animals. RNA was reverse transcribed into cDNA by using the Transcriptor First Strand cDNA synthesis kit from Roche Diagnostics and was analysed by real-time quantitative PCR on ABI 7500 Fast sequence detection system (PE Biosystems). Primer were designed with Primer Express 3.0 (Applied Biosystems) and synthesized by Biotez. Sequences are listed in table S1. We analyzed the heart for brain natriuretic peptide (BNP) and connective tissue growth factor (CTGF), and the kidney for CTGF, neutrophil gelatinase-associated lipocalin (NGAL) and nephrin.

All data are presented as means ± SEM. Statistically significant differences in mean values were tested by ANOVA and the Tukey's multiple range tests. A value of p<0.05 was considered statistically significant.

## Results

**Figure 1:**
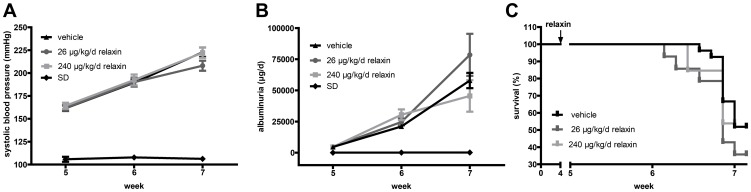
Effect of relaxin on blood pressure, urinary albumin excretion, and survival. (A) Systolic blood pressure increased progressively in vehicle-treated dTGR from week 5 to week 7. Relaxin treatment had no influence on blood pressure; SD rats were normotensive. (B) Vehicle-treated dTGR showed significantly increased 24-hour urinary albumin excretion compared with control values. Relaxin did not eliminate the development of albuminuria in dTGR. (C) Kaplan-Meier survival analysis of vehicle-treated dTGR and dTGR rats receiving low and high dose relaxin. By week 7, half of untreated dTGR were dead. Relaxin did not improve survival of the dTGR. No controls died before end of study (data not shown).

Systolic blood pressure increased progressively in vehicle-treated dTGR from 162±3 mm Hg in week 5 to 225±5 mm Hg in week 7 (figure 1A). Relaxin treatment did not reduce blood pressure (low dose 208±6 mm Hg and high dose 222±6 mm Hg at week 7, respectively). SD rats were normotensive (106±1 mm Hg). Furthermore treatment with relaxin (low dose 78.5±16.8 mg/day and high dose 43.6±10.8 mg/day at week 7) did not ameliorate albuminuria compared to vehicle-treated dTGR (57.937±6.122 mg/day at week 7) (figure 1B). Vehicle-treated and relaxin treated dTGR showed increased urinary albumin excretion compared to SD (0.14±0.06 mg/day at week 7). Finally, relaxin treatment did not reduce mortality (figure 1C). Survival was 52%, in vehicle-treated dTGR, it was 36% in low dose and 54% high dose relaxin treated dTGRs at week 7. Treatment with recombinant human relaxin led to serum concentrations that were between 600 pg/ml and 1150 pg/ml after three weeks of treatment. We detected no human relaxin in vehicle-treated dTGR (data not shown).

**Figure 2:**
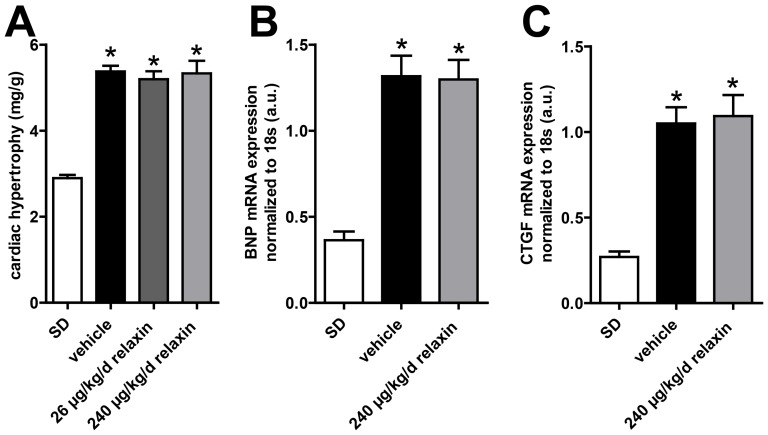
Effect of relaxin on cardiac hypertrophy, BNP and CTGF expression. Treatment with relaxin did not attenuate the development of cardiac hypertrophy (A) expressed as ratio of heart weight to body weight. Cardiac hypertrophy indices of vehicle-treated and treated dTGR rats were significantly higher compared with nontransgenic SD rats. With RT-PCR, we examined BNP (B) and CTGF (C) mRNA expression in the heart. BNP and CTGF mRNA expression was significant higher in vehicle-treated dTGR compared to SD rats. There were no differences in mRNA expression between untreated and relaxin-treated dTGRs. mRNA levels of the target genes were normalized for the housekeeping gene 18S. Results are expressed as mean ± SEM of at least 5 animals per group.

**Figure 3:**
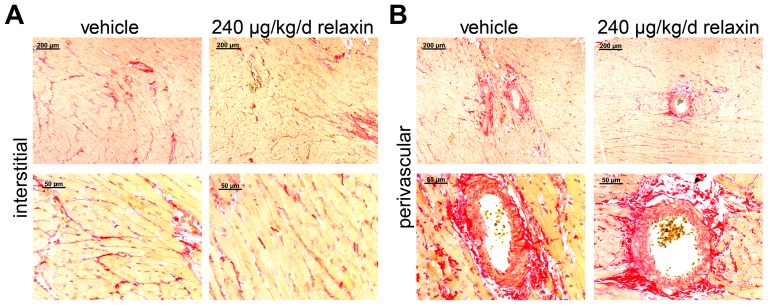
Cardial perivascular and interstitial fibrosis. Representative Sirius red stained heart section from a vehicle-treated and a relaxin-treated dTGR rat. Heart sections of relaxin-treated dTGR showed the same interstitial (A) and perivascular (B) matrix deposition compared to vehicle-treated dTGR animals. The upper panel shows a higher magnification.

Cardiac hypertrophy index (heart-to-body weight) of vehicle treated (5.38±0.13 mg/g) and relaxin-treated dTGR (low dose 5.20±0.18 mg/g and high dose 5.33±0.29 mg/g, respectively) were significantly higher than in SD rats (2.90±0.08 mg/g) (figure 2A). Body weights of vehicle-treated and relaxin-treated dTGR were not different. However, SD rats were heavier than dTGR (data not shown). BNP and CTGF mRNA expression was higher in vehicle-treated dTGR compared to SD rats (figure 2B and 2C). There were no differences in BNP mRNA expression between vehicle-treated and relaxin-treated dTGR. To examine the extracellular matrix deposition, we used histological analysis of Sirius red stained heart sections. Interstitial cardiac fibrosis was present in heart sections of vehicle-treated dTGR rat (figure 3A). Furthermore, Sirius red staining showed large stained areas around the vessels of hearts from vehicle-treated dTGR indicating perivascular cardiac fibrosis (figure 3B). Heart sections of relaxin-treated dTGR showed the same interstitial and perivascular cardiac fibrosis compared to vehicle-treated dTGR.

**Figure 4:**
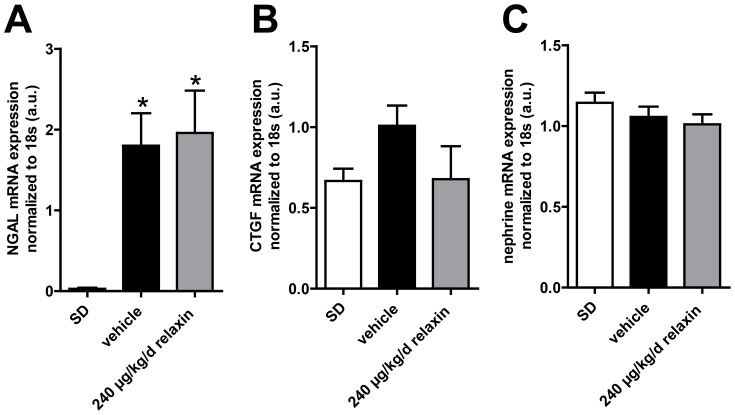
NGAL, CTGF and nephrine expression in the kindney. NGAL mRNA expression (A) was significant higher in vehicle-treated dTGR compared to SD rats. There were no differences in NGAL expression between vehicle-treated and relaxin-treated dTGR. CTGF (B) and nephrin (C) mRNA expression showed no significant changes between SD rats, vehicle-treated and relaxin-treated dTGR. mRNA levels of the target genes were normalized for the housekeeping gene 18S. Results are expressed as mean ± SEM of 5 animals per group.

**Figure 5:**
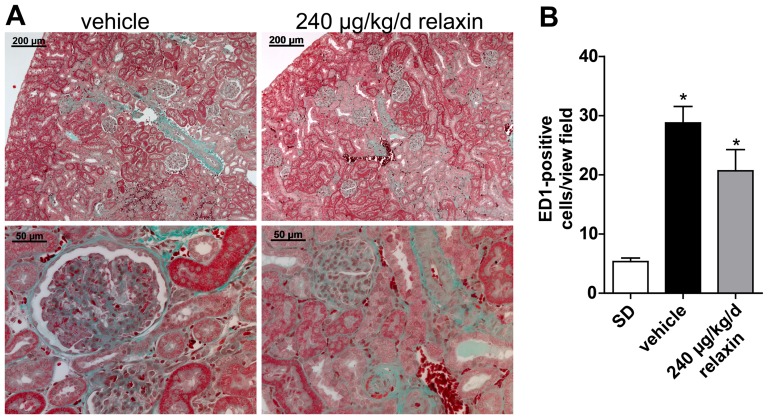
Renal fibrosis and inflammation. Masson trichrome staining of kidney sections (A) showed increased matrix deposition in vehicle-treated dTGR. Relaxin did not reduce matrix formation in the kidney (representative images). The upper panel shows a higher magnification. (B) Semi-quantification of ED-1-positive cells in the kidney revealed that relaxin did not reduce monocyte/macrophage infiltration in kidney. At least fifteen different areas of each kidney were analyzed. Results are mean ± SEM of 4 animals per group.

**Figure 6:**
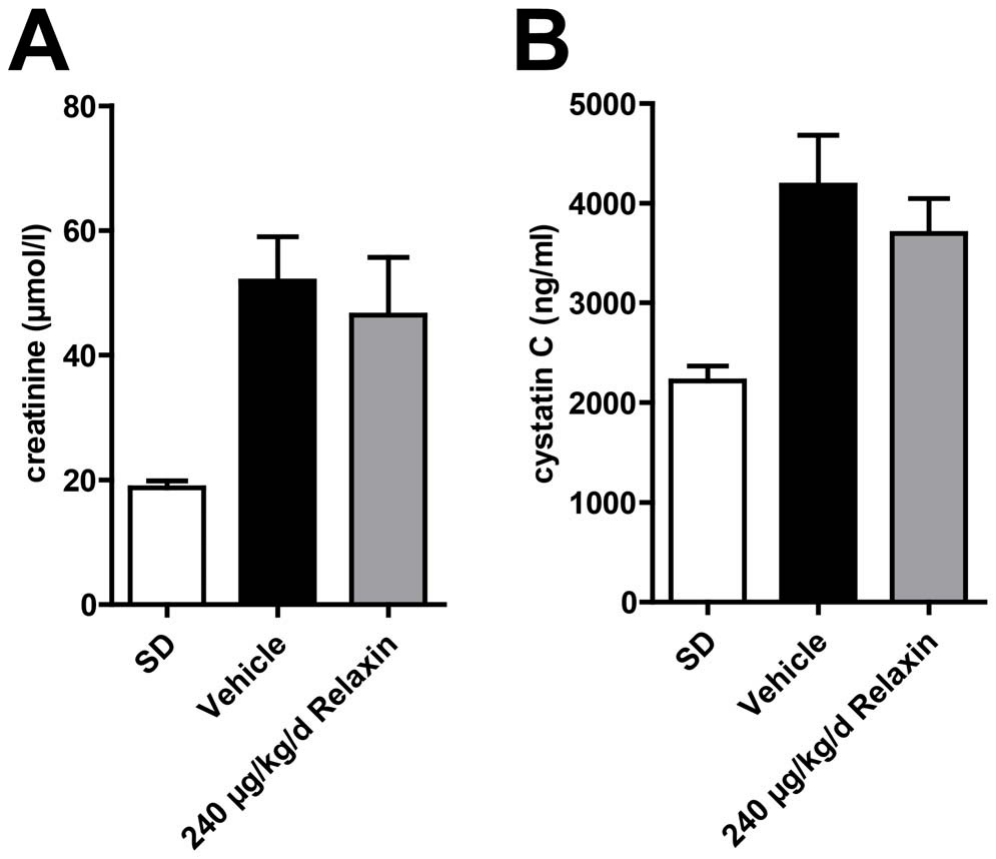
Creatinine and cystatin C serum concentrations. Serum creatinine in vehicle-treated dTGR (A) was significantly elevated at week 7 compared with SD. There were no differences in serum creatinin concentration between vehicle-treated and relaxin-treated dTGR. (B) Serum cystatin C in vehicle-treated dTGR was significantly increased at week 7 compared with SD rats. Relaxin treatment did not alter serum cystatin C concentration in dTGR animals. Results are expressed as mean ± SEM of at least 7 animals per group.

NGAL mRNA expression was significantly higher in vehicle-treated dTGR compared to SD rats (figure 4A). There was no difference in NGAL mRNA expression between vehicle-treated and relaxin-treated dTGR. CTGF and nephrin mRNA expression showed no significant changes between SD rats, vehicle-treated and relaxin-treated dTGR (figure 4B and 4C). Masson trichrome staining in the kidneys revealed that vehicle-treated dTGR had increased matrix deposition (figure 5A). This matrix deposition was also observed in kidney sections of relaxin-treated dTGR. Renal macrophage and monocyte infiltration (ED-1 positive cells) was significantly increased in untreated dTGR compared with SD rats (figure 5B and S1). Again, relaxin treatment did not prevent local macrophage and monocyte infiltration in the kidney. Serum creatinine (figure 6A) and cystatin C (figure 6B) were significantly increased in dTGR compared to controls. Both parameters were not altered after administration of relaxin.

## Discussion

Relaxin treatment had no influence on blood pressure, urinary albumin excretion, or mortality in the dTGR model of hypertension-induced target-organ damage. Furthermore, no improvement of cardiac hypertrophy and reduced matrix formation, as well as connective tissue growth factor expression, was observed in the heart of relaxin-treated dTGR animals. Relaxin also failed to protect the kidney from Ang II-induced damage, as indicated by the fact that relaxin did not ameliorate albuminuria and NGAL expression, renal fibrosis, and inflammation. Most likely relaxin did not improve the endorgan damage in this model, since blood pressure, inflammation as well as profibrotic pathways in kidney and heart were not improved by relaxin.

Relaxin has been evaluated in several acute heart failure (AHF) trails in different cohorts of patients [6], [7]. Pre-RELAX-AHF was a double blind, multicenter, placebo-controlled randomized dose-ranging study of relaxin in 234 AHF patients, enrolled within 16 h after presenting with AHF [7]. Although not powered for endpoints, marked improvement in dyspnea and resolution of systemic and venous congestion was more observed in the group receiving relaxin 30 μg/kg/day for 48 h. There was a significant reduction in 180-day cardiovascular mortality in the relaxin group (3% vs. 14.3% in the placebo group).

The inclusion criteria and definition of AHF were the same in the RELAX-AHF study, as in the PRERELAX-AHF study [6]. The 1,161 subjects had a mean systolic blood pressure of 142 mmHg. Relaxin resulted in a greater SBP reduction both during and for 24 h after infusion. Consistent with previous findings from PRERELAX AHF, there was a significant reduction in cardiovascular death and all-cause mortality at 180 days in the relaxin-treated group. Hernandez-Montfort studied patients with ischemic heart disease and reduced ejection fraction. Their patients were normotensive or hypertensive and had moderate renal impairment [23].

The dTGR model has nothing in common with these clinical studies. The severely hypertensive dTGR develop diastolic heart failure and cachexia during young adulthood. The model is one of fulminant target-organ damage [13]. The threshold dose of recombinant human relaxin to achieve an increase renal function in rats was 0.15 μg/h when given by subcutaneous osmotic minipump [24]. The infusion rate of our low dose relaxin treatment was 0.2 μg/h. This was close to the threshold dose and therefore maybe at the lower therapeutic range. However high dose of relaxin treatment had a 2 μg/h infusion rate, which should be in an efficient range. Several studies were performed with a higher dosage (4 μg/h) for a shorter duration (5–14 days) [25]–[29]. Since the dTGR rats at 3 weeks only weight around 90 g, the highest concentration we could achieve was 2 μg/h. We cannot exclude that a higher dosage was able to reduce cardiovascular endorgan damage in this model. Furthermore we cannot exclude that an earlier start of relaxin therapy has a positive effect on endorgan damage. The dTGR pathology depends on the effects of circulating and local Angiotensin II. Although we have shown in earlier studies that anti-inflammatory therapies ameliorate renal and cardiac endorgan damage, the effects can only be transferred tot he human situation with caution. The pathology of the dTGR and the accelerated speed of the developing endorgan damage are unique for this model.

The different pathways modulated by relaxin suggested that the substance could be beneficial, especially the potent antifibrotic properties exhibited by relaxin [2], [30]. Relaxin inhibited fibroblast proliferation, differentiation, collagen synthesis, collagen deposition, and increased MMP-2 expression, which most likely contributed to an increase in collagen degradation and a decrease in collagen deposition [2], [30], [31]. However, probably the hypertension and the Ang II-induced inflammatory response are the major hallmarks for the target-organ damage and stimulation of the relaxin pathway was not sufficient to break this vicious circle [15]. Beyond that the dTGR model is associated with impaired endothelium-mediated vascular relaxation, whereas endothelium independent vascular relaxation is unchanged [22]. Although endothelial nitric oxide synthase (eNOS) expression in heart and aorta were unchanged compared to controls (data not shown), endothelial dysfunction might be due to uncoupled eNOS. Several studies have shown that relaxin effects are endothelium and NO dependent [20], [32]. Thus, impaired endothelium mediated vascular relaxation in the dTGR model could explain the missing relaxin effectiveness.

Recently Parikh et al. showed beneficial effects of relaxin on atrial fibrillation in spontaneously hypertensive rats (SHR). Relaxin treatment reversed the transcripts for fibrosis, increasing conduction velocity, reduced electrophysiological abnormalities, and reversed atrial hypertrophy [33]. In their study a one-week therapy was ineffective in suppressing atrial fibrillation (AF) and longer relaxin treatment was necessary. The authors speculate that reversal of fibrosis is a slow process as a result of the slow collagen turnover rate of around 5% per day in healthy hearts. The fibrosis in our dTGR is much more pronounced [15]. This state-of-affairs and the fact that fibrosis accelerates over time might be the reason why relaxin was not successful in ameliorating target-organ damage in dTGR [15].

We were also interested in the kidney in dTGR. Recently Yoshida et al demonstrated that relaxin protected against ischemia/reperfusion-induced renal injury by reducing apoptosis and inflammation [29]. Relaxin also preserved renal function in their model. Their relaxin dose, namely 500 ng/h was higher than our high-dose. Moreover, the design of their study was based on acute renal failure; our study was chronic in nature. Parikh et al did not investigate inflammation, while Yoshida et al showed that the TNFα receptor-1 was up regulated in the kidney and normalized by relaxin [29], [33]. We found no such effects in dTGR model. The transcription factors nuclear factor-κB and activator-protein 1 are strongly activated and responsible for the sustained inflammatory and proliferative response in this model [18], [34]. Macrophages, dendritic cells and CD4 and CD8 T cells are strongly activated. Surface adhesion molecule expression, such as ICAM-1, VCAM-1, TNFα and interleukin-6, tissue factor production, and activation of enzymes producing reactive oxygen species are highly induced [13], [17], [19]. We speculate that relaxińs anti-inflammatory potential was not sufficient to counteract the pro-inflammatory storm in dTGR.

In contrast to our findings, Lekgabe et al. showed that relaxin reduced target-organ damage in spontaneous hypertensive rats [35]. However, end-organ damage takes 9–10 months to develop in that model and is far less severe than the effects reported here. Relaxin, applied for 2 weeks, normalized fibrosis in heart and kidney, inhibited cell and increased MMP-2 expression. Blood pressure was not affected by relaxin treatment and mortality was not investigated.

Wong et al investigated the effects of relaxin on fibrosis in streptozotocin (STZ)-treated transgenic mRen-2 rats. That model is also Ang II-mediated and features fairly severe changes. Relaxin did not ameliorate glomerulopathy in this accelerated model of type 1 diabetes [36]. Relaxin did not reduce hypertension or albuminuria. Similar to our study, the authors were puzzled by the negative results. The authors observed that relaxin was very successful in reversing fibrosis when the TGF-β1 pathway was activated and subsequently phosphorylation and down regulation of smad-2 occurred [37]. This was not the case in the dTGR. We noted earlier that protein expression levels of TGF-β were not significantly upregulated in the dTGR [34]. By immunohistochemistry, we showed that TGF-β remained unchanged in the neointima and media of arterial blood vessels, as well as to infiltrated cells perivascular and between cardiomyocytes.

Relaxin did not improve survival in a mouse model of coronary artery ligation [38]. The two time-points chosen in that study represented the important phases of the early and mature fibrotic healing process. Although relaxin significantly inhibited the progression of cardiac fibrosis in the mouse model, cardiac function including fractional shortening was not improved. Similarly, Xu et al investigated the effect of endogenous relaxin on the development of cardiac hypertrophy, dysfunction, and fibrosis after pressure overload using aortic constriction [39]. *Rln* gene-deficient mice showed similar deterioration of cardiac dysfunction and hypertrophy within 8 weeks after chronic pressure overload, compared to *Rln* control mice.

We observed that relaxin did not alter blood pressure. This has already been observed in SHR rats and after Ang II infusion [28]. In a short term experiment over 6 hours relaxin was able to increase cardiac output and significantly decreased systemic vascular resistance without changing mean arterial pressure in both hypertensive rat models. The renal damage in dTGR is most severe in the juxtamedullary cortex, leading to a direct dependence on perfusion pressure [40]. This injury reduces the autoregulation of renal blood flow which impairs an important protective mechanism for the glomerulus [41]. Pressure variations cannot be buffered, leading to further injury of the glomerular capillaries with glomerulosclerosis and proteinuria. Relaxin increases renal vasodilation and hyperfiltration in the rat by reducing the myogenic reactivity in small renal arteries [31]. Relaxin promotes renal plasma flow and glomerular filtration rate, thereby blunting the renal circulatory response to Ang II [42]. However, if hypertension persists and is not reduced, the protective effects of relaxin could be harmful, leading to progressive loss of autoregulation and increased pressure injury in glomeruli [43]. Nevertheless the group of Conrad has convincingly shown renal autoregulation remained intact in pregnant rat although relaxin inhibited myogenic constriction of renal interlobar arteries [44].

## Conclusion

Recombinant relaxin is an exciting new avenue in clinical medicine and could offer new therapeutic advances in patients with AHF. Relaxin may provide a novel therapy for preeclampsia. However, we could not show that relaxin is capable of reversing Ang II-related effects in a fulminant model of Ang II-induced target-organ damage.

## Supporting Information

Figure S1 **Immunohistochemistry of kidney tissue for ED1-positive cells from SD, untreated and relaxin treated dTGR rats.** ED1, a marker of monocyte/macrophage infiltration, showed prevalent inflammation in untreated dTGRs. Relaxin treatments reduced monocyte/macrophage in-filtration in the kidney.
(TIF)

Table S1 **Primer sequences used for RT-PCR.**
(DOC)
