# Supplementary figures and images for: Relaxin Does Not Improve Angiotensin II-Induced Target-Organ Damage
Source: PLoS One. 2014 Apr 7;9(4):e93743. doi: 10.1371/journal.pone.0093743 (PMC3977876; doi:10.1371/journal.pone.0093743)

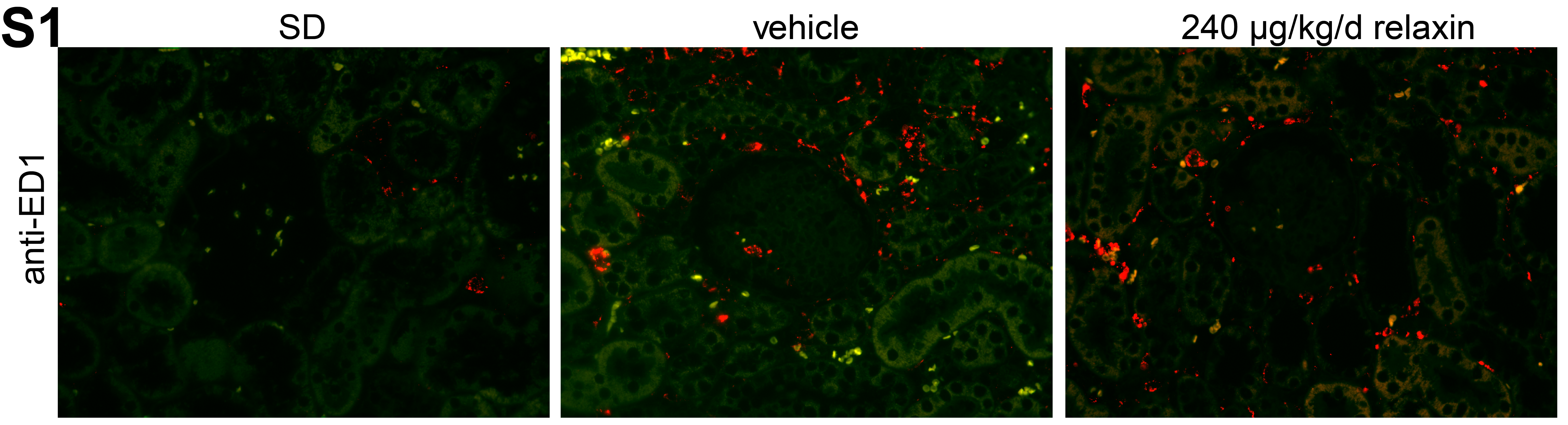

Supplement: Figure S1 — Immunohistochemistry of kidney tissue for ED1-positive cells from SD, untreated and relaxin treated dTGR rats. ED1, a marker of monocyte/macrophage infiltration, showed prevalent inflammation in untreated dTGRs. Relaxin treatments reduced monocyte/macrophage in-filtration in the kidney. (TIF) [file pone.0093743.s001.tif]
