# Supplementary material for: Relaxin Does Not Improve Angiotensin II-Induced Target-Organ Damage
Source: PLoS One. 2014 Apr 7;9(4):e93743. doi: 10.1371/journal.pone.0093743 (PMC3977876; doi:10.1371/journal.pone.0093743)
Supplement: Table S1 — Primer sequences used for RT-PCR. (DOC) [file pone.0093743.s002.doc]

Table S1. Primer sequences used for RT-PCR.

| Gene |  | Sequences (5’→3’) |
| --- | --- | --- |
| BNP | for | CAAGCTGCTTTGGGCAGAAG |
|  | rev | AAACAACCTCAGCCCGTCAC |
|  | probe | AGACCGGATCGGCGCAGTCAGTCGCTT |
|  |  |  |
| CTGF | for | CGCCAACCGCAAGATTG |
|  | rev | CACGGACCCACCGAAGAC |
|  | probe | CACTGCCAAAGATGGTGCACCCTG |
|  |  |  |
| NGAL | for | CAGGGCAGGTGGTTCGTT |
|  | rev | AGCGGCTTTGTCTTTCTTTCTG |
|  | probe | TCGGCCTGGCAGCGAATGC |
|  |  |  |
| Nephrine | for | CAAAAATGTATCACACCAAAGGACAA |
|  | rev | AACACAATCCTGAGGCACAGTCT |
|  | probe | AAGGTTCTGTTTGTCTCCGGTCA |
|  |  |  |
| 18S | for | ACATCCAAGGAAGGCAGCAG |
|  | rev | TTTTCGTCACTACCTCCCCG |
|  | probe | CGCGCAAATTACCCACTCCCGAC |
